# Dataset of intrusion detection alerts from a sharing platform

**DOI:** 10.1016/j.dib.2020.106530

**Published:** 2020-11-17

**Authors:** Martin Husák, Martin Žádník, Václav Bartoš, Pavol Sokol

**Affiliations:** a Institute of Computer Science, Masaryk University, Czech Republic; b CESNET, Czech Republic; c Faculty of Science, Pavol Jozef Šafárik University in Košice, Slovakia

**Keywords:** Cyber security, Intrusion detection alerts, Information exchange, Geolocation, Reputation

## Abstract

The dataset contains intrusion detection alerts obtained via an alert sharing platform (SABU) for one week. A plethora of heterogeneous intrusion detection systems deployed across several organizations contributed to the sharing platform. The alerts are stored in the intrusion Detection Extensible Alert (IDEA) format and categorized using the eCSIRT.net Incident Taxonomy. Dataset can be used in several areas of cybersecurity research for the analysis of intrusion detection alerts including temporal and spatial correlations, reputation scoring, attack scenario reconstruction, and attack projection. The network identifiers (e.g., IP addresses, hostnames) are anonymized. However, the list of interesting features (e.g., presence on blacklists, geolocation) of such entities at the time of data collection is provided.

## Specifications Table

**Table:** 

Subject	Computer science
Specific subject area	Cybersecurity, intrusion detection.
Type of data	Intrusion detection alerts and auxiliary information on the IP addresses included in the alerts.
How data were acquired	The alerts were collected via various intrusion detection systems and honeypots deployed in multiple computer networks.
Data format	RAW. The main data file contains alerts in Intrusion Detection Extensible Alert (IDEA), auxiliary files contain additional information on the IP addresses in CSV files, geographical information are also available as a shapefile for geographic information systems.
Parameters for data collection	The data set contains intrusion detection alerts, i.e., traces of cyber attacks observed by intrusion detection systems, honeypots, and other sensors in computer networks. The data were collected in a distributed environment by a plethora of heterogeneous sensors, which allows for observation of cyberattacks from multiple perspectives that complement each other.
Description of data collection	The data were collected for one week, from March 11 to March 17, 2019, in the SABU alert sharing platform that allows sharing intrusion detection alerts between organizations. Almost 12 million alerts were collected from intrusion detection systems, honeypots, and other data sources deployed in the computer networks of three distinct organizations. Collected alerts contain over 1.7 million IP addresses enriched by their geolocation, presence on blacklists, data from internet-wide scanning and from the Passive DNS system. The alerts were cleansed and anonymized to prevent a leak of sensitive data.
Data source location	The data were collected in three computer networks in the Czech Republic, including the National Research and Education Network (NREN), a campus network, and a commercial Internet Service Provider (ISP) network.
Data accessibility	Repository name: Mendeley Data - Dataset of intrusion detection alerts from a sharing platform Data identification number: http://dx.doi.org/10.17632/p6tym3fghz.1 Direct URL to data: https://data.mendeley.com/datasets/p6tym3fghz/1
Related research article	M. Husák, V. Bartoš, P. Sokol, A. Gajdoš, Predictive Methods in Cyber Defense: Current Experience and Research Challenges, Future Generation Computer Systems. 15 (2021) 517-530. doi:10.1016/j.future.2020.10.006

## Value of the Data

• The data set represents a unique collection of data gathered from heterogeneous distributed intrusion detection systems that complement each other in the detection of large-scale network security incidents [1], [2], [3], [4]. The data allow a deeper understanding of current attack patterns and the behavior of the adversaries from multiple perspectives.
• The data set serves as a reference for researchers to evaluate and compare their approaches to the analysis of intrusion detection alerts, especially in a distributed or collaborative environment, which lacks such data sets [1,5]. The data set offers an up-to-date view of network security alerts and reflects the current cybersecurity threat landscape.
• The data set encourages experimenting with the advanced methods of alert aggregation and correlation [4], including temporal and spatial correlations [6], reputation scoring [7], attack scenario reconstruction [8], and attack projection [9].
• Alert correlation and attack scenario reconstructions methods allow inferring insights into the behavior of the attackers. Such knowledge helps tailor novel attack detection and projection and incident response mechanisms that are effective against current cyber threats. It is vital for cybersecurity to update data sets and traces of attacks as new ones appear continuously [5].
• Temporal and spatial correlations allow for characterizing the overall cybersecurity situation and its changes in time [6]. Reputation scoring helps in assembling effective blacklists [7]. Both approaches enable the prioritization of incidents during the incident handling process, which improves cybersecurity capabilities.
• Modern approaches to alert correlation, attack scenario reconstruction, and attack projection often rely on data mining and machine learning [5] and, thus, it is vital to have a set of training data that resemble real-world scenarios. The presented data set allows such training.

## 1 Data Description

The dataset consists of the main file with the intrusion detection alerts and four auxiliary files with enriched data. The alerts were collected from the SABU alert sharing platform^1^ for one week and are stored in the IDEA format^2^. IDEA is based on and extends the well-known and widely used IDMEF (Intrusion Detection Message Exchange Format)^3^. IDEA adds a taxonomy of alerts (based on eCSIRT.net Incident Taxonomy^4^) and uses modern JSON format instead of XML. A converter^5^ is available for users who wish to work with IDMEF. Almost 12 million alerts were collected from 34 intrusion detection systems, honeypots, and other data sources deployed in three distinct organizations. The IP addresses, hostnames, URLs, and other identifiers in the alerts are anonymized, but the data in the auxiliary files allow profiling malicious actors. The auxiliary files contain data on over 1.7 million IP addresses found in the alerts, the most frequent identifiers of attackers and victims of observed events. Geolocation, data from the Passive DNS system, and other enrichment data are provided. The enrichment includes information on the presence of the IP addresses on publicly available blacklists or outputs of scans by Internet-wide scanners. The geolocation provides the approximate geographical locations of the IP addresses; a data layer for a common geographical information system is provided. The Passive DNS data are in the form of a feature vector of domain names the IP addresses were translated to in the time of their involvement in malicious activities.

In the first subsection, we describe the main data file with the intrusion detection alerts. In the following subsections, we describe the auxiliary materials that consist of spatial data, Passive DNS records, and other enrichment.

### 1.1 Intrusion detection alerts

**Fig. 1:**
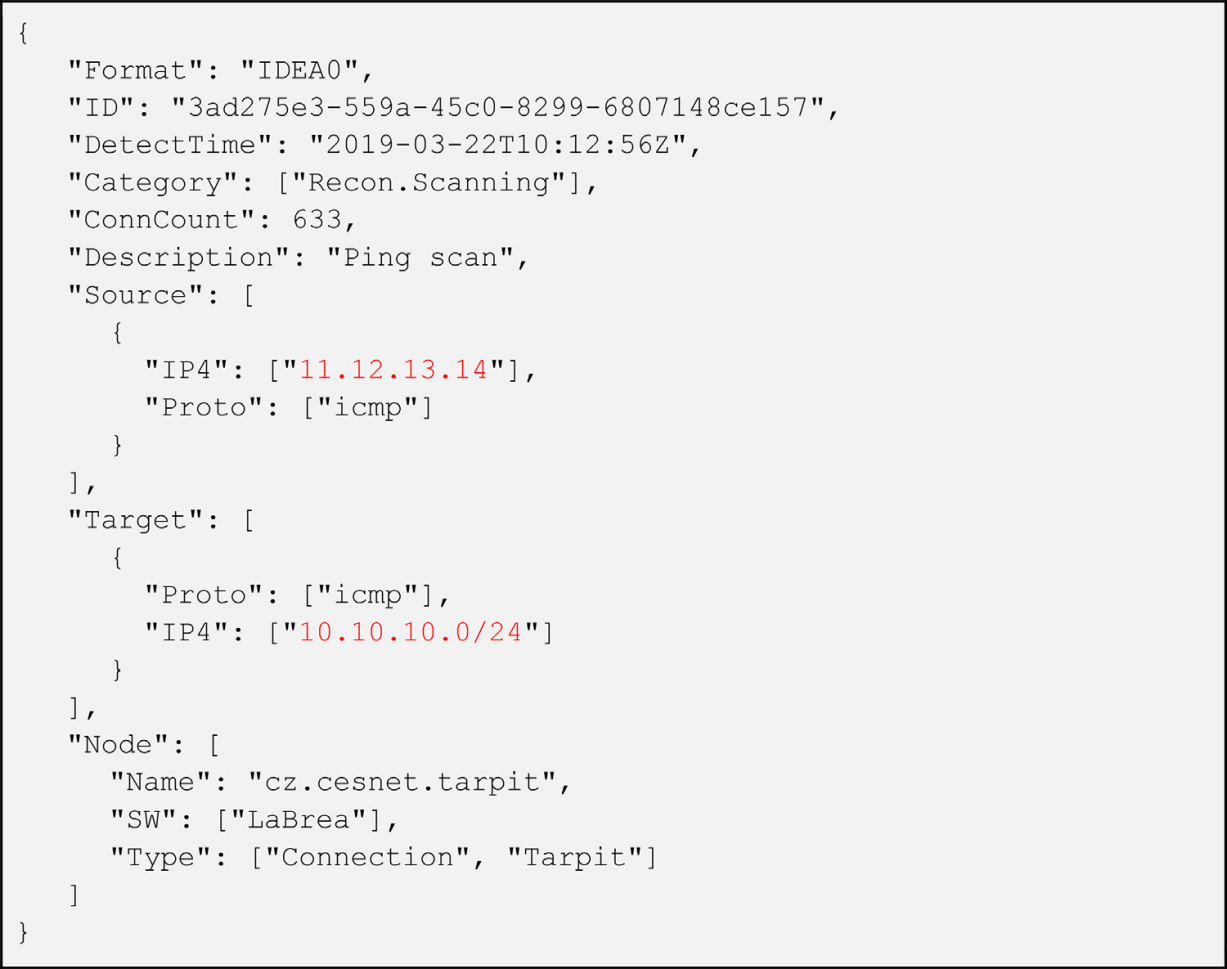
Example of an alert in IDEA format as exchanged in SABU, anonymized entries are red.

The main file *dataset.idea* (compressed in *dataset.idea.zip* archive) contains intrusion detection alerts, one per line sorted in chronological order by the time of arrival in the sharing platform. An example of an alert is presented in Fig. 1. We collected almost 12 million alerts from 34 distinct data sources deployed in 3 organizations: national research and education network (NREN), a large campus network, and a commercial Internet service provider (ISP). Most of the alerts were raised by honeypots and network-based intrusion detection systems based on NetFlow [10], such as NEMEA [11].

### 1.2 Spatial data

The first two auxiliary files contain spatial data. Each IP address in the main dataset was enriched with the spatial data. For this purpose, we used online geospatial service ip-api^6^ and R language^7^ with rgeolocate package^8^. In total, 1,738,062 unique non-anonymised IP addresses have been enriched with the following spatial data:

• geographical coordinates (latitude and longitude),
• country,
• region,
• city,
• time zone,
• Autonomous System Number (ASN),
• Internet Service Provider (ISP).

The output of the enrichment process is a CSV file (*Aux_1A_Geolocation.csv*) containing anonymized IP addresses and spatial data on the original IP addresses (1,738,062 records with 9 variables) separated by a semicolon.

In addition, we prepared a shapefile for the analysis in the geographic information systems (GIS) that are available in the file *Aux_1B_GIS_data.zip*. For this purpose, we used the geographic information system ArcGIS 10.6^9^. As an input, we used the geolocation data cleared of 157 records within geographical coordinates (using the function “Add data” in ArcGIS) and have created a geographic layer (using function “Display XY Data” in ArcGIS) with following settings:

• X field - “Lon” column in CSV file,
• Y field - “Lat” column in CSV file,
• Z field - none, and
• coordinate System of Input Coordinates - World - WGS_1984 (WKID: 4326 Authority: EPSG).

**Fig. 2:**
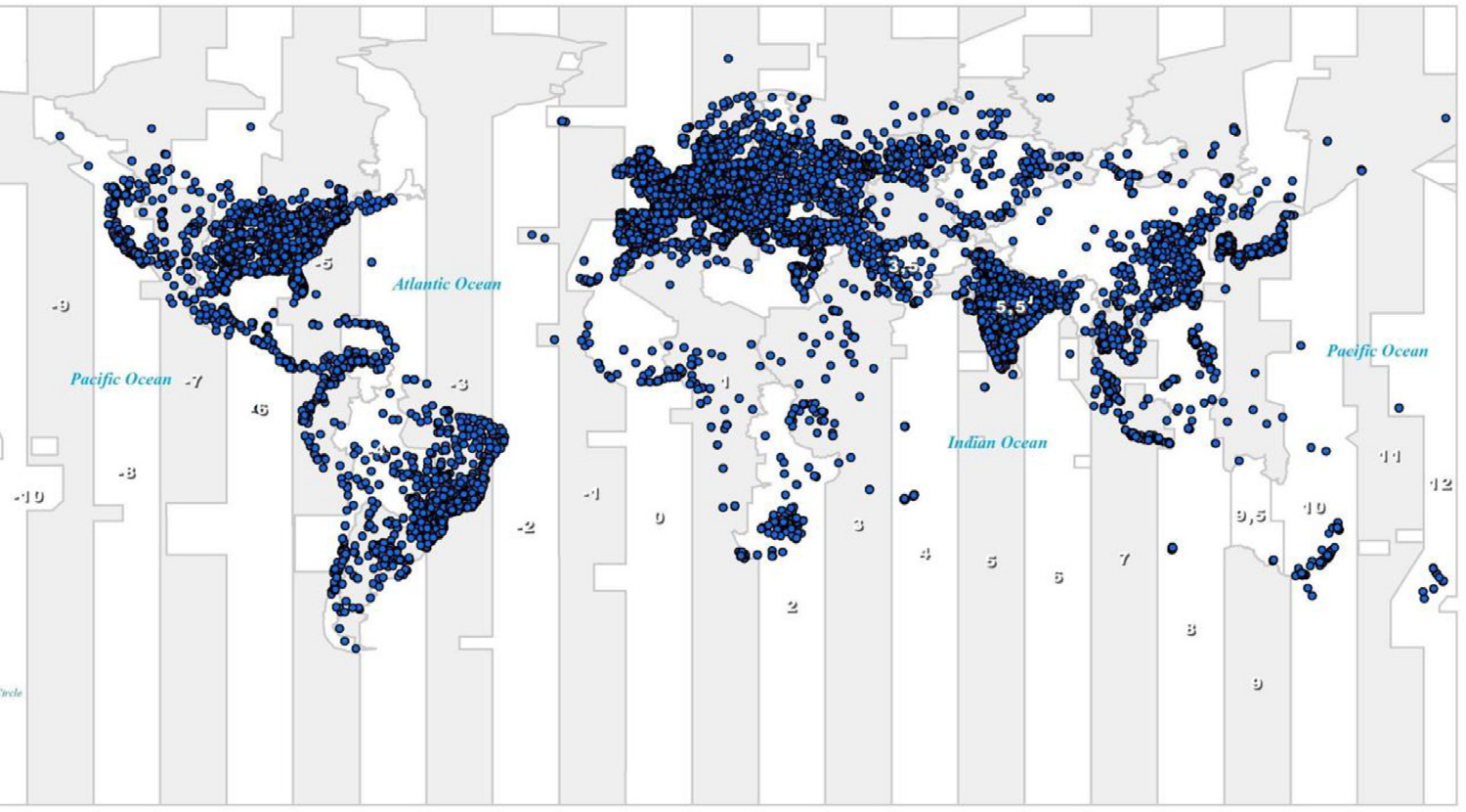
Map of locations of IP addresses in the dataset as a GIS layer.

Furthermore, we exported spatial data as a GIS layer (using the function “Data / Export Data” in ArcGIS) with all features. We provide the transformation of spatial data from one coordinate system to another. In ArcGIS ArcToolbox: Data management tools / Projections and transformations / Project can be used. For a lot of GIS tools (e.g., Kernel density), it is needed to transform data layers from the original geographic coordinate system ("on a 3D ellipsoid", e.g., WGS184) into a projections coordinate system, e.g., Web Mercator Auxiliary Sphere based on WGS 1984 (this system is used by Google Maps). In this way, the original data are geometrically transformed into a planar cartesian coordinate system with metric distance units. The original data in the WGS184 geographic coordinate system can be used, e.g. by Kernel density tool, but it must be explicitly specified in the tool settings that the distance units are geodesic (i.e., degrees). An example of a GIS layer is presented in Fig. 2. Each blue point represents IP addresses with a similar geographical location.

Finally, we have saved spatial data as shapefile, which is a simple, nontopological format for storing the geometric location and attribute information of geographic features^10^. A shapefile is one of the spatial data formats for GIS systems and consists of the following files (packaged in *Aux_1B_GIS_data.zip*):

• IP_GIS.shp - the main file that stores the feature geometry,
• IP_GIS.cpg - an optional file that can be used to specify the code page for identifying the character set to be used (in our case UTF-8),
• IP_GIS.dbf - the database file that stores the attribute information of features.
• IP_GIS.prj - the textual file that stores the coordinate system information,
• IP_GIS.sbn - the file containing that store the spatial index of the features,
• IP_GIS.sbx - the file containing that store the spatial index of the features,
• IP_GIS.shx - the index file that stores the index of the feature geometry.

### 1.3 Passive DNS data enrichment

The second file with auxiliary data is *Aux_2_PassiveDNS.csv* that contains information on DNS records. Each IP address was enriched with the domain names it is associated with utilizing Passive DNS database. Passive DNS^11^ is able to answer what domain names is an IP address associated with by passively listening to DNS resolution and storing its outputs. Since presenting the domain names could compromise the anonymity of data only statistics (mean, standard deviation, maximum, median) on the domain names are provided per each IP address if there is an existing Passive DNS record. The full list of features is as follows:

• number of domain names,
• statistics on the number of levels in the domain names,
• statistics on lengths of whole domain names,
• statistics on the similarity of whole domain names,
• statistics on the entropy of whole domain names,
• statistics on consecutive characters in whole domain names,
• statistics on lengths of lowest-level domain names,
• statistics on the similarity of lowest-level domain names,
• statistics on the entropy of lowest-level domain names,
• statistics on consecutive characters in lowest-level domain names.

**Fig. 3:**
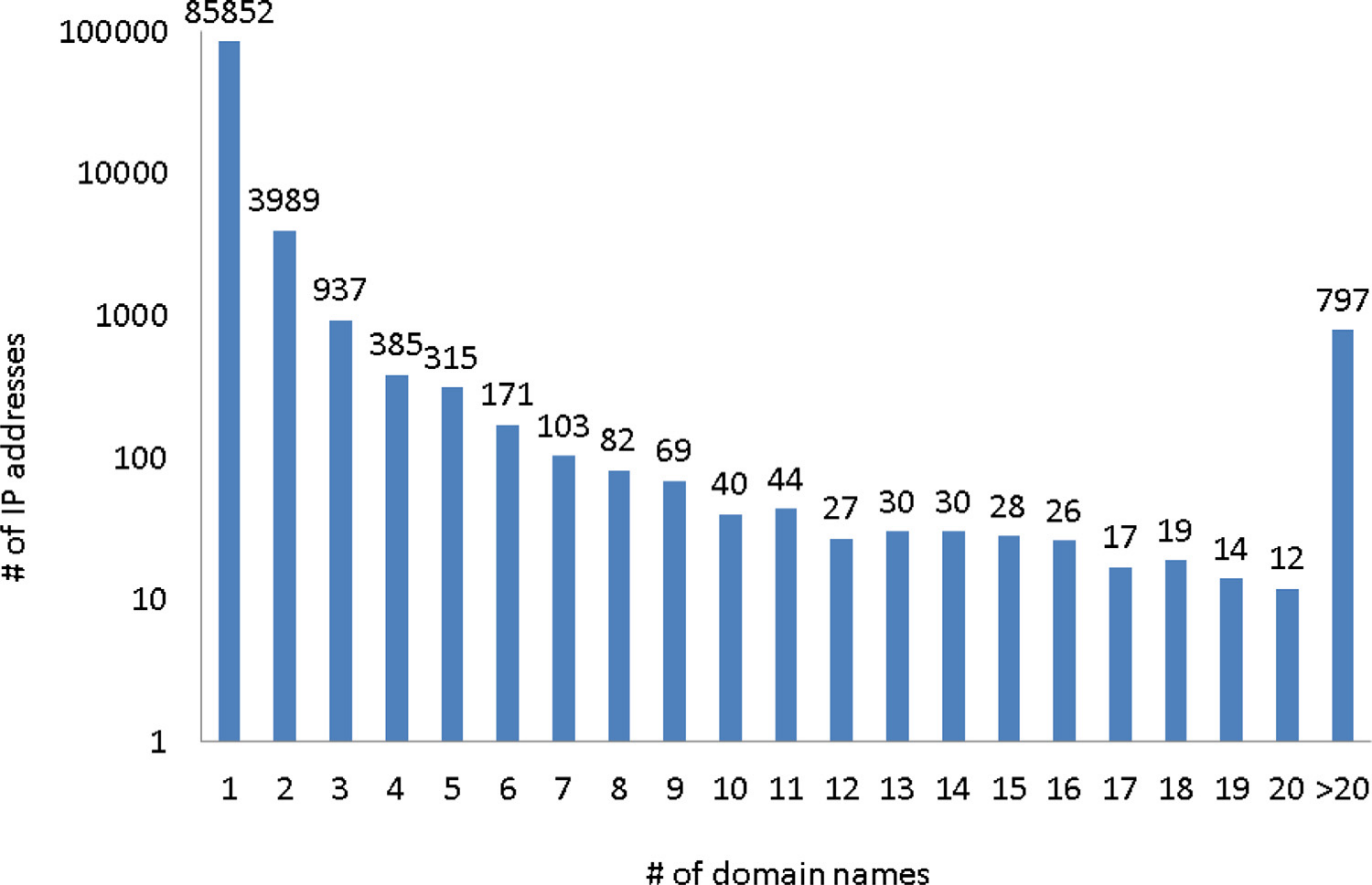
Histogram of number of domain names per IP address.

Fig. 3 shows the number of domain names per IP address as captured by Passive DNS. The majority of IP addresses have either none or one domain name. The maximum number of domain names a single IP address in the data set is associated with is 394,018.

### 1.4 Other enrichment

The third file with auxiliary data is named *Aux_3_Enrichment.zip*, which contains several files with actual data in TSV format. Data in TSV (tab separated values) contain auxiliary data about all IP addresses observed in the dataset. The first line contains column labels. Missing (unknown) values are represented by "-". Hostnames are outputs of DNS PTR queries (using linux "host" command) run for all IP addresses shortly after the dataset capture period.

The Censys fields come from the Internet scanning service of the same name^12^ [12]. Daily snapshots of the Censys database (generously provided to us for free for research purposes) were used. For every day, all IP addresses occurring in the dataset were queried in the matching snapshot, and the outputs were saved. The following fields of the Censys database were queried: IP addresses, ports, protocols, tags, and metadata.

Files *censys_YYYYMMDD.json* contain raw outputs of these queries in JSON format. Since they may contain some additional information, we included them as well. These JSON files were processed by the *censys_json_to_tsv.sh* script into TSV files, which contain selected information in serialized form. These were then joined with the other data, thus creating *ip_data_YYYYMMDD.tsv* files.

For each IP address in the TSV files, tags derived from hostname are always the same for all days (since the hostnames were queried only once), presence on blacklists and Censys data are specific for each day.

The features of the enriched data are stored in the columns of the TSV files. The features can be categorized into three groups, and the full list of columns in the data files is as follows. The first group of features specifies whether the IP address was present on a given blacklist at a given date. The features are:

• tor - list of Tor exit nodes^13^,
• blocklist_de_ssh - blacklist of SSH attackers from blocklist.de^14^,
• uceprotect - UCEPROTECT-level1 blacklist^15^,
• sorbs-dul - "dial-up" blacklist by SORBS (queried using DNSBL at dnsbl.sorbs.net),
• sorbs-noserver - "noserver" blacklist by SORBS (queried using DNSBL at dnsbl.sorbs.net),
• sorbs-spam - "spam" blacklist by SORBS (queried using DNSBL at dnsbl.sorbs.net),
• spamcop - Spamcop blacklist (queried using DNSBL at bl.spamcop.net),
• spamhaus-pbl - Spamhaus Policy Block List (ISP maintained) (queried using DNSBL at zen.spamhaus.org),
• spamhaus-pbl-isp - Spamhaus Policy Block List (Spamhaus maintained) (queried using DNSBL at zen.spamhaus.org),
• spamhaus-xbl-cbl - Spamhaus Exploits Block List (queried using DNSBL at zen.spamhaus.org).

The second group of features contains hostname tags, i.e., several properties of the IP addresses guessed from their associated hostnames (PTR record):

• hostname_exists - 1 if PTR DNS query returned a valid response, 0 otherwise,
• dynamic_static - 1 if the IP address is dynamically assigned, -1 if it's static, 0 otherwise; determined by matching the hostname against the following regexes: *'\bdyn(amic)?|pool|dial-?up\b', '\bstatic\b',*
• dsl - whether it looks like a DSL connection; regex: *'\b(broad(band)?|[avx]dsl|cable)\b'*
• vpn - whether it's part of a VPN; regex: '\bvpn[0-9]*\b',
• nat - whether it's public IP of a NAT device; regex: '\bnat[0-9]*\b',
• ip_in_hostname - whether the hostname is automatically generated from the IP, i.e., at least the last two octets of the IP address are found as numbers in the hostname.

Finally, the third group of features contains the data from Censys.io database, i.e., list of open ports and services and information extracted from service banners (look into Censys.io documentation for details about how the data are gathered):

• censys_protocols - which TCP ports/protocols are open/available on the IP address,
• censys_tags - tags assigned by the Censys.io service,
• censys_device_type - inferred device type,
• censys_product - Product name,
• censys_os - Operating system running on the host,
• censys_os_version - Operating system version.

## 2 Experimental Design, Materials and Methods

This section is structured in two subsections. First, we describe the SABU alert sharing platform, in which the data were collected. Subsequently, we describe the collected data and their sanitization in more detail.

### 2.1 SABU alert sharing platform

The data were collected for the period of one week, from March 11 to March 17, 2019, in the SABU alert sharing platform that allows sharing intrusion detection alerts between organizations. Readers are kindly referred to selected surveys [1], [2], [3] for the list and comparison of similar alert sharing platforms and collaborative intrusion detection systems. SABU was developed and deployed in Czech Republic, where the data collection was conducted. Moreover, software components, services, and data formats originating in SABU were also used by European research projects PROTECTIVE^16^ and researchers from 8 european countries, and the interest in the platform persists. The central point of SABU is Warden^17^, a system that receives alerts sent by various intrusion detection systems, honeypots, and third-party sharing platform, and distributes them to various recipients, such as analytical tools (e.g., iABU^18^), reporting tools (e.g., Mentat^19^), and active network defense devices. The data can be enriched by the reputation database NERD^20^. NERD was developed alongside SABU and, as of 2020, has its own public interface and serves an active user base from 10 countries excluding Czech Republic.

The three organizations that deployed the honeypots and intrusion detection systems contributing to the SABU platform are covering three different networks. Further, two of the networks overlap, which allows for interesting situations for alert correlation. The network of NREN is a backbone network geographically distributed around the Czech Republic. The campus network is in a single geographic location but connected to the NREN network. Thus, the network-based intrusion detection systems of the NREN can also oversee the network traffic of the campus network. The network, commercial ISP, is also geographically distributed, but the network does not overlap with the other two.

The NREN network deployed the most intrusion detection systems and honeypots. The most significant is the network-based intrusion detection system NEMEA [11] that detects port scans, brute-forcing, DDoS attacks, DDoS amplifiers, communications with IP addresses on blacklists, and anomalies in network traffic. The alerts from NEMEA can be found under the node name cz.cesnet.nemea.*, where * stands for a detection method. Alternative network-based intrusion (e.g., Suricata^21^, *cz.cesnet.ids_collector.suricata*) and anomaly (e.g., FTAS, *cz.cesnet.ftas*) detection systems are also present, although with a lesser number of reported alerts. Some networks of the NREN also use the TippingPoint^22^ intrusion prevention systems; the alerts from TippingPoint can be found under the node name *cz.cesnet.ids_collector.tippingpoint*. There are also various honeypots and tarpits deployed in the NREN network and reporting to SABU platform, such as cowrie^23^ (*cz.cesnet.hugo.haas_cowrie*), dionaea^24^ (*cz.cesnet.hugo.haas_dionaea*), LaBrea^25^ (*cz.cesnet.tarpit*), and others. The honeypots report network scans, brute-forcing, and exploitation attempts. Finally, various third-party data sources are contributing to SABU via connectors deployed in the NREN network. The node names share the name format cz.cesnet.ext.*.

The campus network operates two network-based intrusion detection systems, NEMEA [11] and Flowmon ADS^26^. The NEMEA instance performs the detection of port scans, brute-forcing, communication with IP addresses on blacklists, DDoS amplifiers, and the detection of anomalies in network traffic. Alerts from NEMEA at the campus network can be found under the node name cz.muni.ics.csirt.nemea.*, where * is the intrusion detection module. Flowmon ADS (node name *cz.muni.ics.csirt.flowmon_ads*) is a commercial intrusion and anomaly detection system that detects similar events, such as port scanning, brute-forcing, and communication with IP addresses on blacklists. Further, there are two data sources that share alerts from honeypots deployed in the campus network. The node *cz.muni.ics.csirt.ackbar* is a machine running Heralding^27^ honeypot that reports brute-forcing, and the node *cz.muni.ics.csirt.honeyscan* is honeyscan, an in-house flow-based honeypot monitoring system that reports network scans and brute-forcing.

Finally, the commercial ISP operates only the NEMEA [11] intrusion detection system. The active intrusion detection modules include port scan detection, brute-forcing detection, DDoS detection, detection of DDoS amplifiers, detection of communication with IP addresses on blacklists, and network anomaly detection. All the data sources have the node name cz.casablanca.nemea.*, where * stands for an intrusion detection module.

### 2.2 Intrusion detection alerts

The alerts were filtered and anonymized, misleading alerts or their parts were deleted to avoid confusion and to prevent an unintentional leak of sensitive data. A detailed description of the process goes as follows.

First, we sanitized and cleansed the data. Although we observed no malformed alerts or alerts that did not conform to the specification of the IDEA definition, there were other cases, where we had to delete the alerts or their parts. Full alerts were deleted only if they were designated as testing alerts or if they included private or dedicated IP addresses. In the first case, the *Category* entry in the alerts has to include tag *Testing*. These alerts are from data sources (intrusion detection systems, honeypots, and others) that are under development or are newly connected to the platform to see if they are reliable or not. The testing alerts are filtered out by default in the SABU platform, and it is not recommended to use them anyway. In the second case, we deleted all the alerts containing IP addresses from private and link-local IP address ranges (10.0.0.0/8, 172.16.0.0/12, 192.168.0.0/16, 169.254.0.0/16, fd00::/8, and fe80::/10). Typically, a network-based intrusion detection system detects anomalous network traffic from or to an IP address from these IP address ranges. However, such network communication happens inside the organization's network and is more likely a symptom of a misconfiguration or another case of false-positive detection. In addition, such alerts would be isolated and not suitable for correlation with other alerts. Thus, we deleted such alerts to avoid distorting any analysis over the data. Nevertheless, both testing alerts and alerts containing private or link-local IP addresses do not stand for more than 1% of the alerts in the original dataset and, thus, their absence is not notable.

Subsequently, we deleted parts of many alerts for two reasons. First, we deleted all the data elements that are not documented in IDEA format definition or not conforming to the classification of the alerts. Such entries were deleted to avoid confusion. For example, many data sources include their internal ID of an alert into the IDEA message. Analytical and reporting tools may include their own data entries, e.g., to mark already processed alerts. Such parts of alerts are only interesting for a particular system or organization, and not understood by other peers in the platform. Thus, their deletion does not cause information loss.

The second reason to delete parts of alerts was that there were data entries that posed a risk of disclosing sensitive information that had to be either anonymized or deleted. This relates to three keys in the IDEA specification, *Description, Note*, and *Attach* (attachment). *Description* and *Note* may contain arbitrary text, mostly to help human readers understand the alert. Short description of the reported event or the data source is the desired content. In practice, however, many alerts used a textual description of the event that included the IP addresses, URLs, hostnames, and other items that had to be anonymized or deleted. Deleting the human-readable description is significantly simpler than anonymizing the content, and all the important pieces of information are present in other parts of the alert. Further, if the reported event was based on an entry in a blacklist, the *Description* or *Note* contained the entry from the blacklist and the blacklist's name. Sharing the content of a blacklist might be restricted by its terms of use and, thus, we had to delete such entries. However, the information on which (anonymized) IP address was present on which blacklist can be inferred from the auxiliary data. Thus, in both cases, there was no information loss. The attachments, on the contrary, were deleted, because of the low relevance of the content and potential problems caused associated with it. The attachments can contain a variety of data, but at the time of collecting the data, they contained only malware binaries captured by honeypots. Sharing and working with malware samples is problematic on its own and there are well-known repositories and databases specialized on malware samples. Malware analysis is out of the scope of potential use cases of our dataset and, thus, we deleted the attachments with malware samples and left only their hashes and identifiers in the alerts. Researchers interested in alerts correlation and other tasks, for which this dataset was created, do not need to work directly with malware binaries. Thus, deleting them does not cause a loss of important data.

Finally, the data were anonymized. The anonymization is needed for IP addresses, hostnames, and URLs that appear in the alerts and might link to particular entities, organizations, and persons. All the IP addresses in the alerts were anonymized using CryptoPAn^28^. This includes both source and target IP addresses on both IPv4 and IPv6 protocols. CryptoPAn allows for prefix-preserving anonymization of the IP addresses, i.e., the IP addresses from the same subnet before anonymization are in the same subnet after anonymization. The implementation of CryptoPAn allows for the anonymization of IPv4 and IPv6 addresses with the same key that we keep secret. The URLs and hostnames were replaced by anonymized strings containing either “URL” or “Hostname” and a unique integer ID. Thus, for every unique hostname or URL, there is a unique placeholder string, which allows for correlating the alerts with the same URLs or hostnames. No emails or other potentially sensitive pieces of information were found in the data.

## Ethics Statement

The data collection was conducted with the consent of the contributors to the SABU alert sharing platform. All potentially sensitive data were deleted or properly anonymized. The dataset does not disclose any information that could be used to connect cybersecurity events with natural persons or organizations.

## Credit Author Statement

**Martin Husák**: Data Curation, Writing - Original Draft, Writing - Review & Editing **Martin Žádník**: Resources, Data Curation, Project administration **Václav Bartoš**: Software, Resources, Data Curation, Writing - Original Draft **Pavol Sokol**: Data Curation, Writing - Original Draft, Writing - Review & Editing, Visualization

## Declaration of Competing Interest

The authors declare that they have no known competing financial interests or personal relationships which have, or could be perceived to have, influenced the work reported in this article.

## Footnotes

1 https://sabu.cesnet.cz/en/start

2 https://idea.cesnet.cz/en/index

3 https://tools.ietf.org/html/rfc4765

4 https://www.trusted-introducer.org/Incident-Classification-Taxonomy.pdf

5 https://github.com/CSIRT-MU/IDEA-IDMEF-Converter

6 http://ip-api.com/

7 https://www.r-project.org/

8 https://cran.r-project.org/web/packages/rgeolocate

9 http://desktop.arcgis.com/en/

10 http://desktop.arcgis.com/en/arcmap/10.3/manage-data/shapefiles/shapefile-file-extensions.htm

11 For example: https://www.circl.lu/services/passive-dns/

12 https://censys.io/

16 https://protective-h2020.eu/

17 https://warden.cesnet.cz/en/index

18 iABU is a named instance of the AIDA framework: https://github.com/CSIRT-MU/AIDA-Framework

19 https://mentat.cesnet.cz/en/index

20 https://nerd.cesnet.cz/

21 https://suricata-ids.org/

22 https://www.trendmicro.com/en_us/business/products/network/intrusion-prevention.html

23 https://github.com/cowrie/cowrie

24 https://github.com/DinoTools/dionaea

25 http://labrea.sourceforge.net/

26 https://www.flowmon.com/en/products/flowmon/anomaly-detection-system

27 https://github.com/johnnykv/heralding

28 https://www.cc.gatech.edu/computing/Networking/projects/cryptopan/

## Footnotes

13 http://torstatus.blutmagie.de/ip_list_exit.php

14 https://lists.blocklist.de/lists/ssh.txt

15 http://wget-mirrors.uceprotect.net/rbldnsd-all/dnsbl-1.uceprotect.net.gz
